# Comparative transcriptome analysis of gene responses of salt-tolerant and salt-sensitive rice cultivars to salt stress

**DOI:** 10.1038/s41598-023-46389-1

**Published:** 2023-11-04

**Authors:** Xin Fang, Junjie Mo, Hongkai Zhou, Xuefeng Shen, Yuling Xie, Jianghuan Xu, Shan Yang

**Affiliations:** 1 College of Coastal Agricultural Sciences, Guangdong Ocean University, Zhanjiang, 524088 China; 2 South China Branch of National Saline-Alkali Tolerant Rice Technology Innovation Center, Zhanjiang, 524088 China

**Keywords:** Plant sciences, Plant molecular biology, Plant physiology, Plant stress responses

## Abstract

Salt stress is one unfavorable factor of global climate change that adversely affects rice plant growth and yield. To identify novel salt-tolerant genes and new varieties of salt-tolerant rice, a better understanding of the molecular regulation mechanism of salt tolerance in rice is needed. In this study we used transcriptome analyses to examine changes in gene expression of salt-tolerant and salt-sensitive rice plants. The salt-tolerant cultivar HH11 and salt-sensitive cultivar IR29 were treated with 200 mM NaCl solution for 0 h, 6 h, 24 h and 48 h at the three leaf stage. Physiological parameters and transcriptome were measured and analyzed after each treatment. Activity of SOD and POD, as well as the MDA and protein content of the two rice cultivars generally increased with increasing time of exposure to NaCl. Meanwhile, the APX activity first increased, then decreased in both cultivars, with maximum values seen at 6 h for IR29 and at 24 h for HH11. The GR and GPX activity of HH11 were stronger than that of IR29 in response to salt stress. The H_2_O_2_ content first increased at 0–6 h, then decreased at 6–24 h, and then increased again at 24–48 h under salt stress. Compared with IR29, SOD, POD and APX activity of HH11 was more sluggish in response to salt stress, reaching the maximum at 24 h or 48 h. The MDA, H_2_O_2_ and proline content of HH11 was lower than that of IR29 under salt stress. Relative to untreated HH11 plants (0 h) and those exposed to salt for 6 h, 24 h, and 48 h (H0-H6, H0-H24 and H0-H48), 7462, 6363 and 6636, differentially expressed genes (DEGs), respectively, were identified. For IR29, the respective total DEGs were 7566, 6075 and 6136. GO and KEGG enrichment analysis showed that metabolic pathways related to antioxidative responses and osmotic balance played vital roles in salt stress tolerance. Sucrose and starch metabolism, in addition to flavonoid biosynthesis and glutathione metabolism, showed positive responses to salt stress. Expression of two *SPS* genes (*LOC_Os01g69030* and *LOC_Os08g20660*) and two *GST* genes (*LOC_Os06g12290* and *LOC_Os10g38740*) was up-regulated in both HH11 and IR29, whereas expression of *LOC_Os09g12660*, a glucose-1-phosphate adenylyltransferase gene, and two *SS* genes (*LOC_Os04g17650* and *LOC_Os04g24430*) was up-regulated differential expression in HH11. The results showed that HH11 had more favorable adjustment in antioxidant and osmotic activity than IR29 upon exposure to salt stress, and highlighted candidate genes that could play roles in the function and regulation mechanism of salt tolerance in rice.

## Introduction

Salt stress is an important abiotic stress that seriously affects plant growth. Rice (*Oryza sativa* L.) is a staple food for more than 50% of the global population and is a moderately salt-sensitive crop^1,2^. Salt stress in rice reduces the germination, growth rates and tillering number that in turn affects biomass and plant height. Decreases in thousand grain weight, panicle number and setting percentage that compromise grain yield are also caused by salt stress. Rice quality is diminished by salt stress as reflected by decreased grain filling and grain quality and composition^3^. Therefore, understanding the molecular mechanism of salt tolerance in rice and exploration of key salt-tolerance genes is important for breeding of robust, salt-tolerant rice varieties.

At a molecular level, salt stress causes ion poisoning, osmosis and oxidation stress^4^. Ion poisoning is generally caused by excessive accumulation of Na^+^ and Cl^-^ under salt stress leading to accumulation of Na^+^ in the cytoplasm that depolarizes the membrane and promotes leakage of K^+^ out of the cell that eventually causes plant death^5^. The potassium transporter *OsHAK1* was shown to promote K^+^ uptake and increase the K^+^/Na^+^ ratio under salt stress, and thus this gene is essential for enhanced salt tolerance in rice^6^. Numerous genes related to salt transport and that regulate salt tolerance have subsequently been identified including *OsHKT1;4*^7^, *OsHKT1;5*^8^ and *OsAKT2*^9^. To adapt to osmotic stress associated with salt stress, plants produce large of amounts of osmotic adjustment substances like proline, betaine, glycerin and sugars and their derivatives^10^. Oxidative stress caused by salt exposure increases activity of antioxidant enzymes like SOD, CAT, POD and APX as well as synthesis of non-enzymatic antioxidant substances like ascorbic acid, glutathione and carotenoids^11^. As such, the mechanism of salt tolerance involves a complex regulatory network comprising multiple metabolic pathways.

The continued development of sequencing technology has facilitated studies that use transcriptome sequencing data to explore metabolic pathways associated with plant responses to salt stress and to identify salt-tolerant candidate genes. Salt tolerance mechanisms in rice have also been explored using transcriptome sequencing technology. A comparative transcriptomic analysis was performed in contrasting two rice genotypes (a susceptible variety Chao 2R and a tolerant variety RPY geng) under salt stress, which found that the expression of normal life process genes in Chao 2R were severely affected under salt stress, but RPY geng regulated the expression of multiple stress-related genes to adapt to the same intensity of salt stress, such as secondary metabolic process, oxidation–reduction process^12^. A previous study used transcriptome sequencing analysis of rice seedings under salt-stressed soil conditions (250 mM NaCl) to show significant effects on carbohydrate and amino acid metabolism and focused on nine genes that had up-regulated expression in response to salt stress^13^. Wang et al. found that rice seedlings exposed to salt stress had differential gene expression for antenna protein, which is involved in photosynthesis, at 48 h of salt stress relative to 0 h, whereas genes involved in glutathione metabolism, pyruvate metabolism, starch and sucrose metabolism, and tyrosine metabolism showed differential expression at 72 h^14^. A candidate gene that mediates salt tolerance, *OsRCI2-8* (*Os06g0184800*), was identified through a combination of linkage mapping and transcriptome profiling analysis^15^. Together, these studies show the value of RNA sequencing technology for identification of critical salt-tolerant metabolic pathways and mining of salt-tolerant candidate genes in plants.

Despite these efforts, the regulatory molecular network of salt tolerance genes in rice remains unclear. Moreover, to our knowledge, there has been no comparative transcriptome analysis of the effect of salt stress on red rice. Here we identified critical salt-tolerant metabolic pathways and promising candidate genes for engineering of salt- tolerant red rice.

## Materials and methods

### Plant materials and experiment design

The salt-tolerant rice Haihong 11 (HH11, red rice genotype) and salt-sensitive rice IR29 (white rice genotype) belonging to *Oryza sativa* L. subsp. Indica were used in this study. Plump seeds were selected and soaked in a 50 °C water bath for 1.5 h and then placed in a 30 °C incubator and soaked for another 24 h. Then the seeds were transferred onto germinating paper, which was kept wet and incubated at 30 °C. Upon germination, seeds having similar growth vigor were planted in a 96-well black hydroponic box filled with ddH_2_O and cultivated in an artificial climate chamber with 75% relative humidity under 18 h/6 h light/dark (light intensity ~ 16,000 LUX) cycle at 28 °C/24 °C. When the seedlings reached the 1-leaf stage, the hydroponic box was filled with Yoshida nutrient solution for rice (Coolaber, Beijing, China). The nutrient solution was renewed every 3 days.

When the seedlings reached the three-leaf stage, the Yoshida nutrient solution was replaced with nutrient solution containing 200 mM NaCl. Seedlings having similar growth vigor were selected and treated for 0 h, 6 h, 24 h and 48 h with 200 mM NaCl. The leaves of the rice seedlings were clipped and collected for transcriptome and physiological analyses at the corresponding treatment time. All samples (24 samples) were frozen in liquid nitrogen and stored at − 80 °C. Three biological replicates were performed for each sample in this study.

### Measurement of physiological parameters

The malondialdehyde (MDA) content, hydrogen peroxide (H_2_O_2_) content, peroxidase (POD) activity, superoxide dismutase (SOD) activity, ascorbate peroxidase (APX) activity, proline content, glutathione reductase (GR) activity and glutathione peroxidase (GPX) activity were determined using the Dionisio-Sese and Tobita^16^, Góth^17^, Chance and Machly^18^, Asada et al.^19^, Nakano and Asada^20^, Bates et al.^21^, Carlberg and Mannervik^22^, and Bengt^23^ methods, respectively.

### RNA sequencing

Total RNA from the 24 samples was extracted using TRIzol reagent (Invitrogen, Carlsbad, CA, USA) according to the manufacturer’s instructions. HH11 and IR29 plants exposed to salt stress for 0 h, 6 h, 24 h and 48 h were named H0/I0, H6/I6, H24/I24 and H48/I48, respectively. The cDNA sequencing libraries were constructed using a NEBNext Ultra RNA Library Prep Kit for Illumina (NEB, USA) following the manufacturer’s recommendations and index codes were added to attribute sequences to each sample. All libraries were sequenced on an Illumina HiSeq X-ten platform by Beijing Biomarker Technologies Co., Ltd. (Beijing, China) using 150 bp paired-end reads (PE 150).

### Transcriptome data analysis

Raw data in a fastq format were first processed using in-house perl scripts. In this step, clean data (clean reads) were obtained by removing low quality reads and reads containing adapter and poly-N from the raw data. At the same time, Q20, Q30, GC-content and sequence duplication level of the clean data were calculated. All downstream analyses were based on high quality clean data. Clean reads from every sample were mapped to the reference genome for the rice cultivar Nipponbare (http://rice.plantbiology.msu.edu) using HISAT2^24^. Raw counts of genes were determined using feature counts^25^. Differentially expressed genes (DEGs) between two samples were identified using DESeq2 with |log_2_fold change|≥ 1 and a false discovery rate (FDR) < 0.05^26^. DEG functions were annotated using the KEGG and GO databases. KEGG pathway analysis of DEGs was performed with BLAST software^27^ and KEGG enrichment was analyzed using KOBAS 2.0 software with p-value < 0.05^28,29^. GO analysis of DEGs was carried out using the R package clusterProfiler^30^. Transcription factors (TFs) among the DEGs were predicted using iTAK^31^ software with PlnTFDB^32^ and PlantTFDB^33^ databases. The comparisons H0 vs. H6, H0 vs. H24 and H0 vs. H48 in the transcriptome comparison analysis of HH11 are referenced as H0-H6, H0-H24 and H0-H48, respectively. Similarly, comparisons of I0 vs. I6, I0 vs. I24 and I0 vs. I48 are referenced as I0-I6, I0-I24 and I0-I48, respectively.

### The fluorescent quantitative real-time PCR (qRT-PCR) verification

To verify the reliability of RNA sequencing results, 12 DEGs were randomly selected from the transcriptome data for qRT-PCR. Based on the coding gene sequences, the RT-qPCR primers were designed using Primer Premier 6.0 software, and β-actin was selected as the internal control gene (Table S1). Hieff qPCR SYBR Green Master Mix (No Rox) reagent from Yesen Biotechnology Co., Ltd. was used for qRT-PCR. The qRT-PCR assays were carried out with a Bio-Rad CFX96 qRT-PCR instrument using a 20 µl reaction volume containing 10 µl SYBR Green Master Mix, 0.4 µl upstream primer, 0.4 µl downstream primer, 1 µl template DNA, and 8.2 µl sterile ultra-pure water. The qPCR data were analyzed using the 2^−ΔΔCt^ quantitative method to determine differences in gene expression^34^. Three independent biological replicates and three technological replicates were used for each sample.

### Data processing and visualization

SPSS 25.0 software was used for variance analysis using Duncan’s multiple comparison method. The bar diagram was plotted using Graphpad Prism 8.0. Venn diagrams, scatter diagrams and heat maps were drawn using BMKCloud tools (https://www.biocloud.net).

### Ethics statement about plant material

Authors comply with the relevant guideline and legislation of China, IUCN Policy Statement on Research Involving Species at Risk of Extinction and the Convention on the Trade in Endangered Species of Wild Fauna and Flora in this study.

## Results

### Phenotypic response of rice seedlings to salt stress

**Figure 1:**
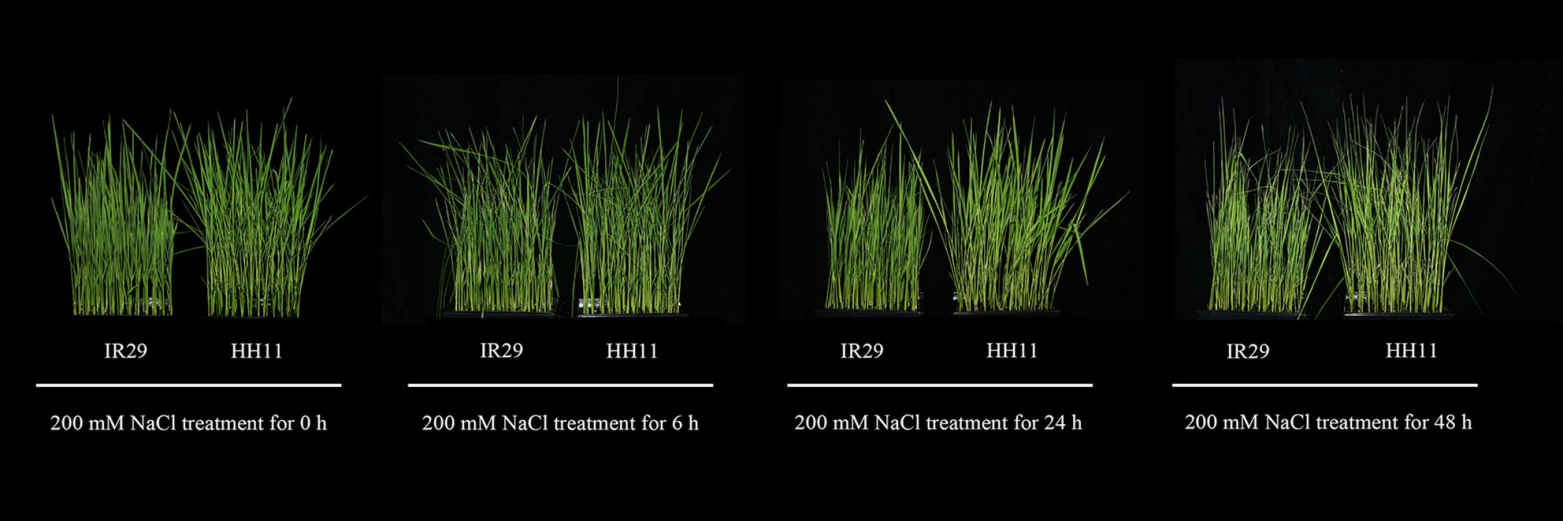
Phenotype of rice seedlings exposed to salt stress for different time periods.

The salt tolerance of the two rice cultivars, the salt-tolerant rice Haihong 11 (HH11, red rice genotype) and a salt-sensitive rice IR29 (white rice genotype), were treated with a high concentration of NaCl solution (200 mM) for 0 h, 6 h, 24 h, or 48 h in the seedling stage. The growth of both types of seedlings was inhibited by salt stress. With increasing time of exposure to salt stress, the leaves of both types of rice plants gradually curled and the leaf tips yellowed, while some stalks sagged or even collapsed (Fig. 1). The salt-sensitive cultivar IR29 was more severely affected than HH11 at 6 h and 24 h, as was manifested by the lower plant height.

### Analysis of physiological parameters under salt stress

**Figure 2:**
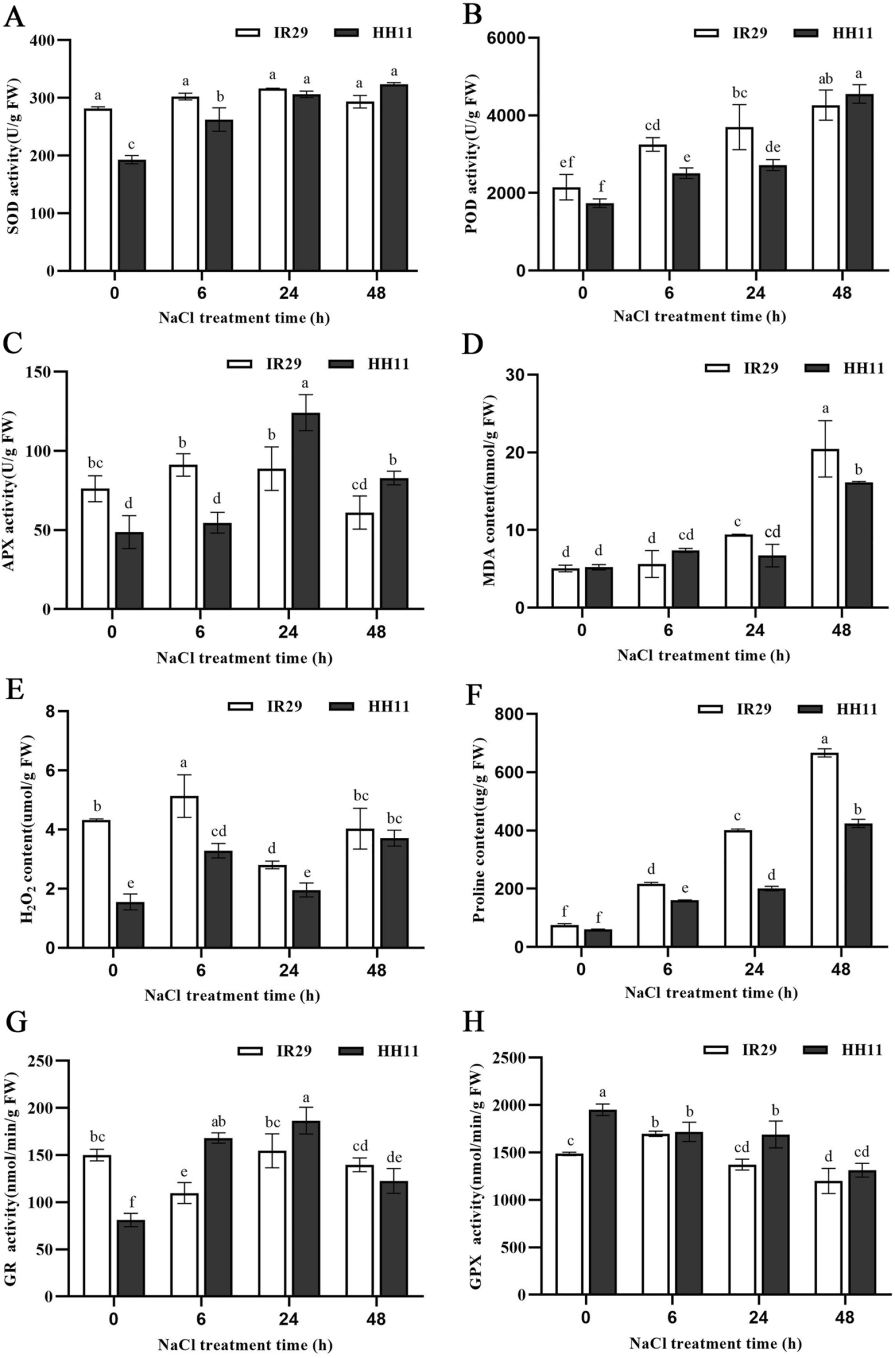
Variation in physiological indexes of rice plants under salt stress. (**A**) SOD activity, (**B**) POD activity, (**C**) APX activity, (**D**) MDA content, (**E**) H_2_O_2_ content, (**F**) Proline content, (**G**) GR activity and (**H**) GPX activity. Error bars represent standard deviation among means for three different samples. Different letters on bars indicate statistically significant differences (*p* < 0.05).

Changes in antioxidant oxidase activity of rice plants exposed to salt were next assessed. In HH11 plants, superoxide dismutase (SOD) activity at 48 h of salt exposure was significantly increased by 40% relative to that of 0 h (Fig. 2A). Meanwhile, compared with 0 h, IR29 SOD activity increased by 11.0% at 24 h, but decreased slightly at 48 h, such that overall there was no significant change in the presence of salt (Fig. 2A). Compared with 0 h, salt exposure increased peroxidase (POD) activity by 98.5% and 162.3% in IR29 and HH11, respectively (Fig. 2B). Ascorbate peroxidase (APX) activity first increased, then decreased in both cultivars, with maximum values seen at 6 h for IR29 (19.7% increase) and at 24 h for HH11 (155.2% increase, Fig. 2C). Together, the increase in antioxidant activity of HH11 was higher than that of IR29 at 48 h of salt stress, and antioxidants in HH11 were activated after 24 h salt stress, but had an overall lower response to salt stress than did the salt-sensitive IR29.

The malondialdehyde (MDA) content of both HH11 and IR29 increased significantly by 3- and twofold, respectively, over 48 h of salt stress (Fig. 2D). IR29 had slightly lower levels of MDA than HH11 at 0 h and 6 h, and significantly higher amounts than HH11 after 24 h. The H_2_O_2_ content of the two cultivars showed a trend of first increasing, then decreasing, and then increasing again, but the H_2_O_2_ content of IR29 was always higher than that of HH11 over the 48 h time period (Fig. 2E). Moreover, there was no significant difference in H_2_O_2_ content between the two varieties at 48 h, but there was significant difference between the two varieties at 0 h, 6 h and 24 h. These results indicate that the degree of lipid membrane damage was higher in IR29 than in HH11.

The proline content of both HH11 and IR29 showed an increasing trend under salt stress (Fig. 2F). The amount of proline in IR29 samples increased by 7.8-fold and HH11 by sixfold by 48 h relative to 0 h. The proline content of IR29 was greater than that of HH11, indicating that IR29 plants was subjected to more severe osmotic stress than HH11. The GR activity of HH11 increased first and then decreased, and reached the maximum value at 24 h during 0–48 h salt stress (Fig. 2G). There were significant differences in GR activity at all stress time points except 6 h and 24 h (Fig. 2G). For IR29, it showed a trend of first increasing, then decreasing, and then increasing again, and there was no significant difference among the treatments (Fig. 2G). There were significant differences in GR activity between the two varieties except that there was no significant difference at 48 h, of which GR activity of HH11 was significantly higher than that of IR29 (Fig. 2G). For GPX activity, it was a gradually decreasing trend with the extension of salt stress time in HH11, and was significant differences at 0 h and 48 h (Fig. 2H). The GPX activity of IR29 increased first and then decreased, and reached the maximum value at 6 h (Fig. 2H). There were significant differences in GPX activity between the two varieties except that there was no significant difference at 6 h and 48 h (Fig. 2H). These results indicate that HH11 has stronger GR and GPX activity than IR29 in response to salt stress.

### Quality control analysis of RNA-seq data

To comprehensively understand the gene expression profile of rice plants under salt stress, sequencing of total RNA was done for 24 samples collected after exposure to 200 mM NaCl treatment for 0 h, 6 h, 24 h or 48 h. To obtain clean data for subsequent analysis, raw data for six transcriptome libraries were filtered by checking sequencing error rates and GC content distribution. A total of 173.92 Gb clean data was obtained. The Q30 for the 24 libraries exceeded 89.61%, whereas the GC content was between 51.19% and 52.84% (Table S2). Clean reads for each sample were mapped with the specified reference genome, and the efficiency of mapping ranged from 81.30 to 90.08% (Table S2). These results indicate that the accuracy of the experimental data was high and was suitable for subsequent bioinformatics analyses (Table S2).

### Differential expression gene analysis

To explore the molecular mechanism of salt tolerance in red rice, differentially expressed genes (DEGs) of plants exposed to salt stress for different time periods was examined. Fold Change ≥ 1 and FDR < 0.05 were used as standards for screening DEGs in response to salt stress. Compared with the control group (0 h), 7462 DEGs (3590 up-regulated and 3872 down-regulated) and 7566 DEGs (3659 up-regulated and 3907 down-regulated) were identified at 6 h for HH11 and IR29, respectively (Table S3). In 24 h, 6363 DEGs (3,001 up-regulated and 3362 down-regulated) and 6,075 DEGs (2800 up-regulated and 3275 down-regulated) were identified for HH11 and IR29, respectively (Table S3). In 48 h, 6636 DEGs (3362 up-regulated and 3274 down-regulated) and 6136 DEGs (3141 up-regulated and 2995 down-regulated) were identified for HH11 and IR29, respectively (Table S3). Thus, for both cultivars the samples collected at 6 h had the highest number of DEGs, indicating that a large number of rice genes are differentially expressed in a short period of time after exposure to salt stress (Table S3). In addition, the number of DEGs for HH11 was always slightly higher than that for IR29 during the same salt stress treatment time, indicating that HH11 had more genes that respond to salt stress (Table S3). The number of up- and down-regulated DEGs was similar between the two plant types with fewer up-regulated DEGs than down-regulated DEGs at 6 h and 24 h, and the opposite trend was seen at 48 h with more down- than up-regulated DEGs (Table S3).

**Figure 3:**
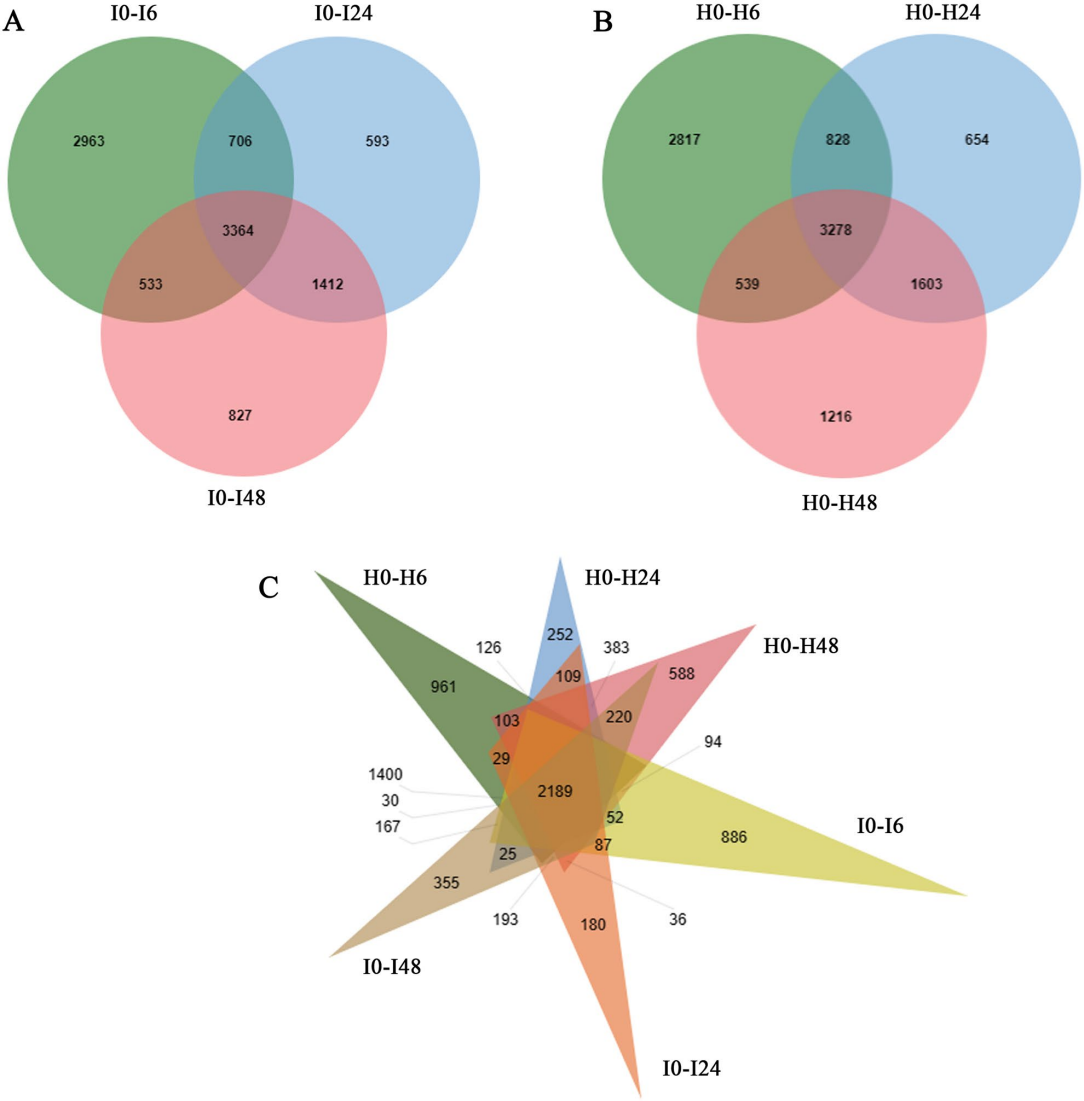
Venn diagrams of DEGs in HH11 and IR29. (**A**) I0-I6, I0-I24 and I0-I48 comparisons for IR29; (**B**) H0-H6, H0-H24 and H0-H48 comparisons for HH11 and (C) Three different compared groups for IR29 and HH11.

We mapped the DEGs of the two plants in a Venn diagram (Fig. 3A, B). For the IR29 comparisons I0-I6, I0-I24 and I0-I48, there were 2,963, 593 and 827 unique DEGs under salt stress (Fig. 3A). These three groups shared 3,364 DEGs (Fig. 3A). For HH11 comparisons H0-H6, H0-H24 and H0-H48, there were 2,817, 654 and 1,216 unique DEGs, respectively (Fig. 3B) and 3,278 DEGs shared by the 3 compared groups (Fig. 3B). HH11 had more unique DEGs than did IR29. A total of 2,189 DEGs appeared in all six comparisons, suggesting that this group may contain key genes that respond to salt stress (Fig. 3C).

### Transcriptome data verified by qRT-PCR

**Figure 4:**
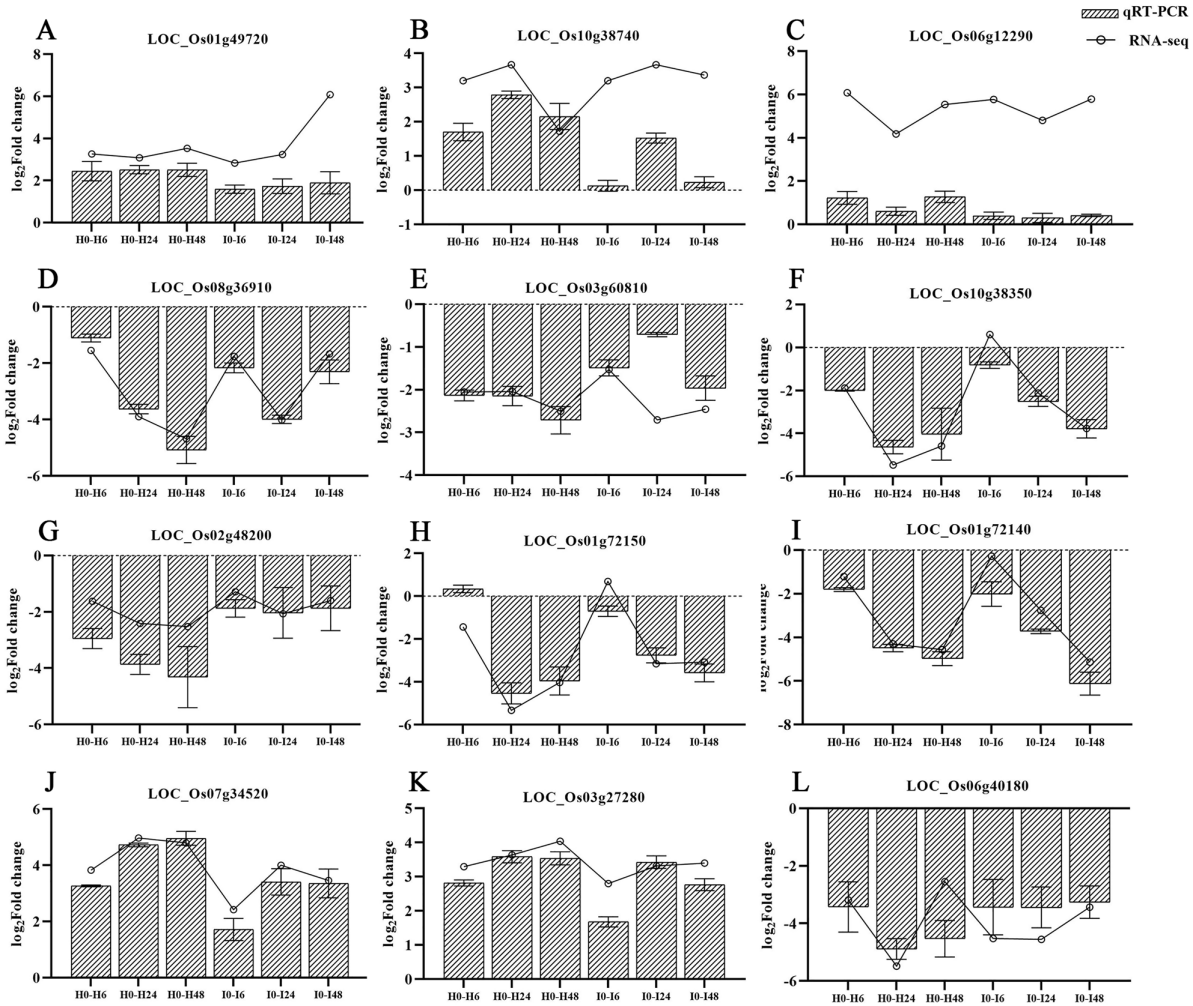
Comparison analysis of qRT-PCR and RNA-seq for 12 genes. The gene expression level of RNA-seq is shown as Log_2_(fold change) and the fold-change is based on FPKM values of the drought stress group relative to the control group. The gene expression level of qRT-PCR is shown as Log_2_(fold change = 2^−ΔΔCt^).

To verify the reliability of transcriptome sequencing data, 12 DEGs were randomly selected for qRT-PCR analysis (Table S1). The trends for up-regulated and down-regulated expression were consistent between RNA-seq and qRT-PCR for all 12 genes (Fig. 4). However, the absolute value of gene expression levels detected by qRT-PCR was less than that for RNA-seq, particularly for *LOC_Os06g12290* (Fig. 4C). These results confirm that there was a similar expression pattern of DEGs detected by qRT-PCR and RNA-seq.

### GO enrichment analysis

**Figure 5:**
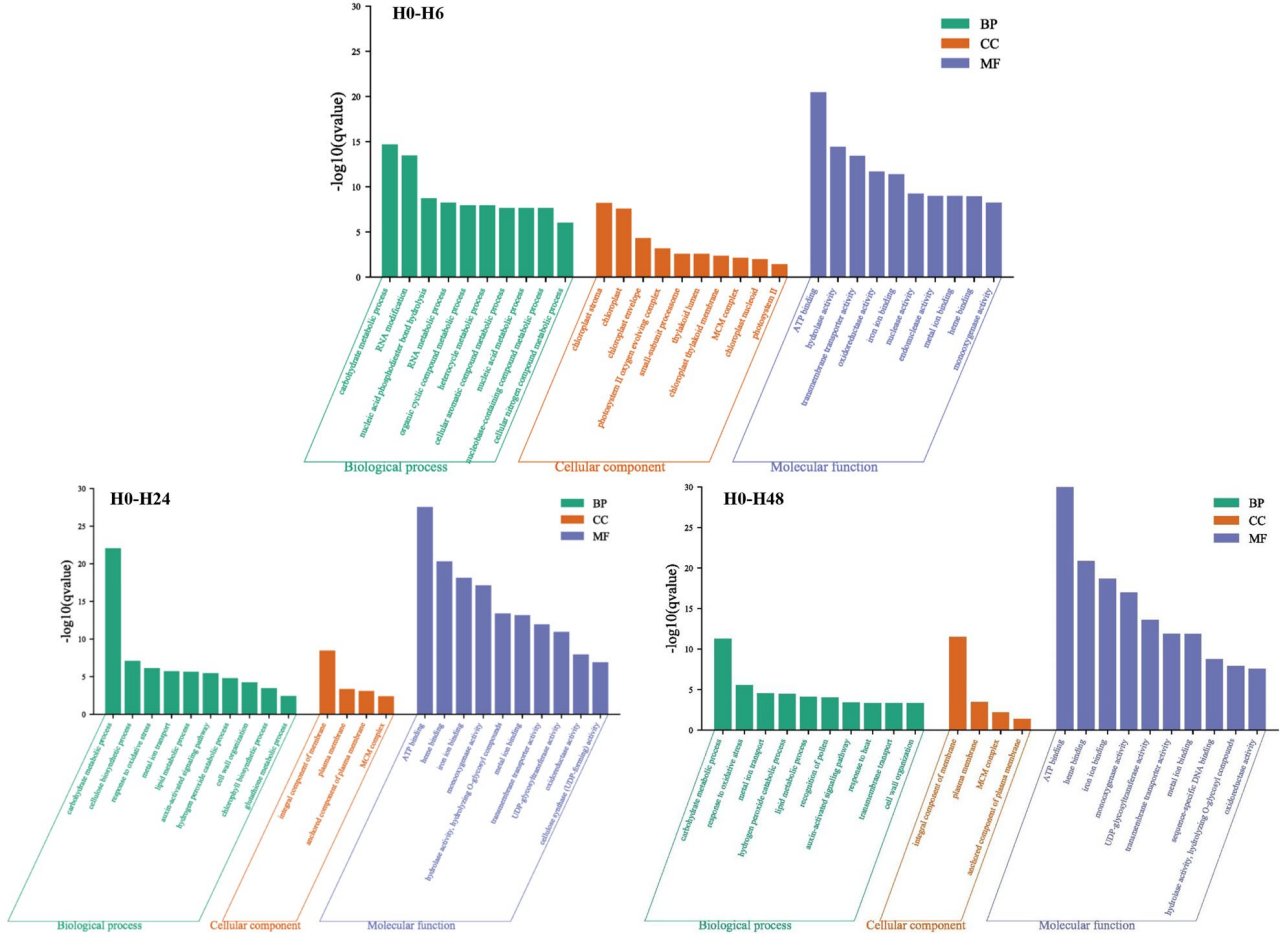
Top-10 GO enrichment terms in HH11 exposed to salt stress for different time periods. BP, CC and MF are short for biological process, cellular component and molecular function, respectively.

**Figure 6:**
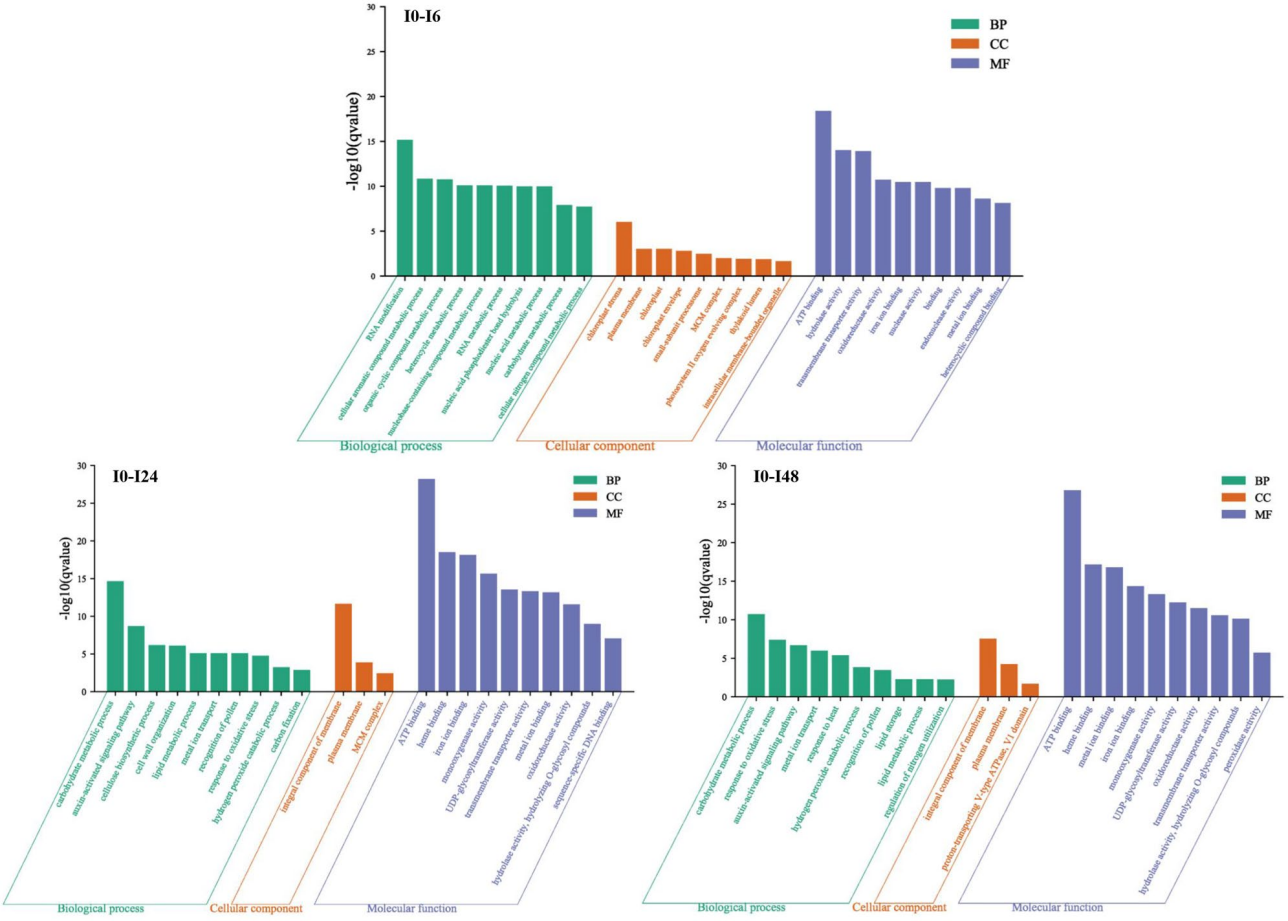
Top-10 GO enrichment terms in IR29 exposed to salt stress for different time periods. BP, CC and MF are short for biological process, cellular component and molecular function, respectively.

GO analysis showed that the number of GO enriched terms was similar for the two rice cultivars (3801, 3288, 3283 enriched for H0-H6, H0-H24, H0-H48, respectively, and 3756, 3105 and 3214 enriched for I0-I6, I0-I24 and I0-I48 groups, respectively (Tables S4 and S5)). For the number of GO terms for respective HH11 comparisons H0-H6, H0-H24 and H0-H48, 2319, 1995 and 1975 were classified as biological process (BP), 439, 370 and 358 were classified as cellular component (CC), and 1043, 923 and 950 were classified as molecular function (MF) (Table S4). For respective IR29 comparisons I0-I6, I0-I24 and I0-I48, 2248, 1869 and 1933 were classified as BP, and 459, 363 and 411 were classified as CC, and 1049, 873 and 870 GO terms were classified as MF (Table S5). To explore specific pathways having GO enrichment, the top-10 GO terms were analyzed for BP, CC and MF components based on q-value < 0.05 (Fig. 5). For BP, carbohydrate metabolic process was at the top of the three compared groups. The hydrogen peroxide catabolic process, metal ion transport, lipid metabolic process, response to oxidative stress, cell wall organization and auxin-activated signaling pathway were present in the H0-H24 and H0-H48 groups (Fig. 5). CC had 4 GO terms shared in the H0-H24 and H0-H48 groups (q-value < 0.05): plasma membrane, integral component of membrane and anchored component of plasma membrane (Fig. 5). In MF, iron ion binding, metal ion binding, transmembrane transporter activity, heme binding, oxidoreductase activity, monooxygenase activity and ATP binding were shared in the H0-H6, H0-H24 and H0-H48 (Fig. 5). Results for IR29 were similar to those for HH11, with carbohydrate metabolic process, response to oxidative stress, oxidoreductase activity, metal ion transport and metal ion binding showing a similar response to salt stress as was seen for HH11 (Figs. 5 and 6).

### KEGG enrichment analysis of differentially expressed genes

**Figure 7:**
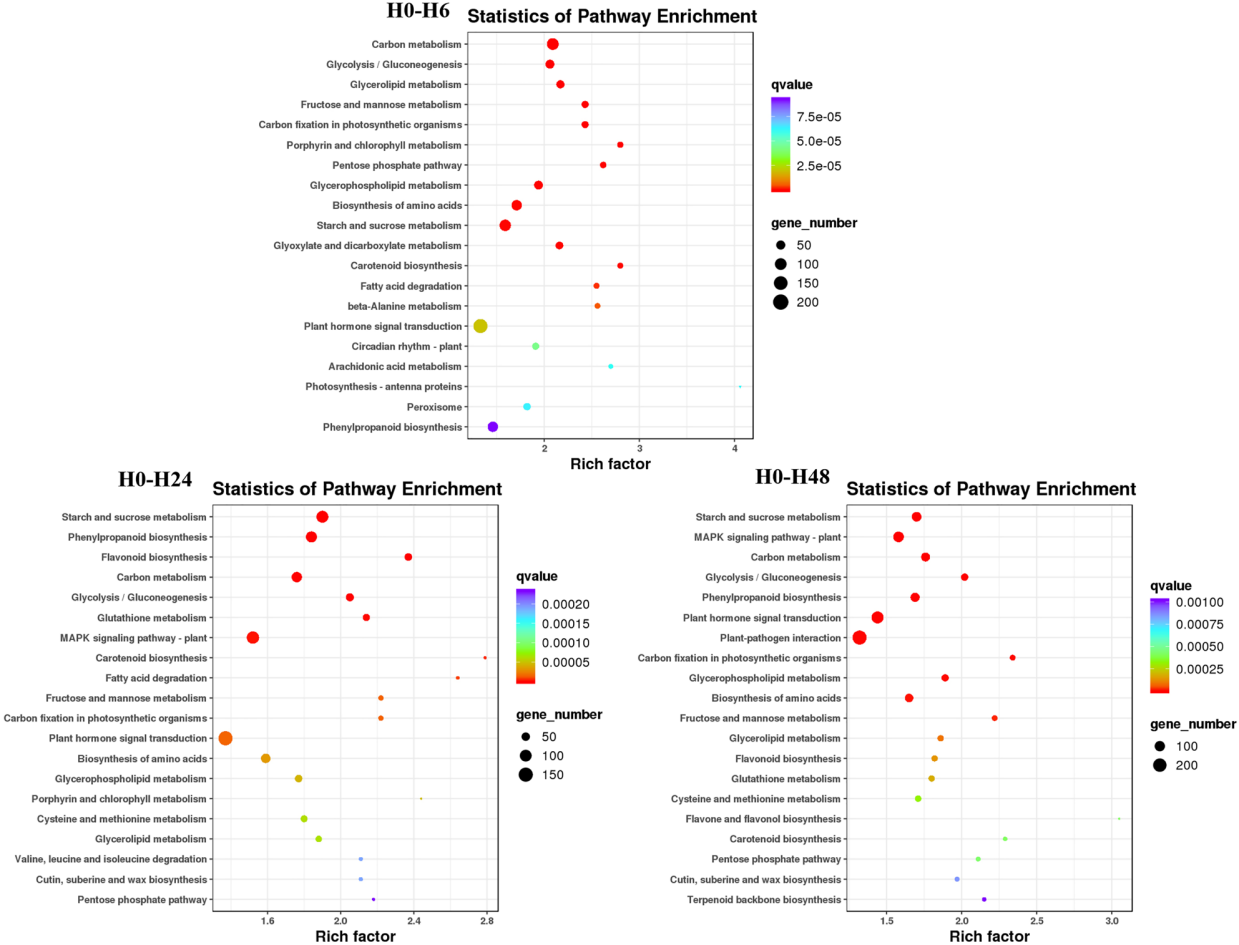
Top-20 KEGG enrichment pathways in HH11 exposed to salt stress for different time periods.

**Figure 8:**
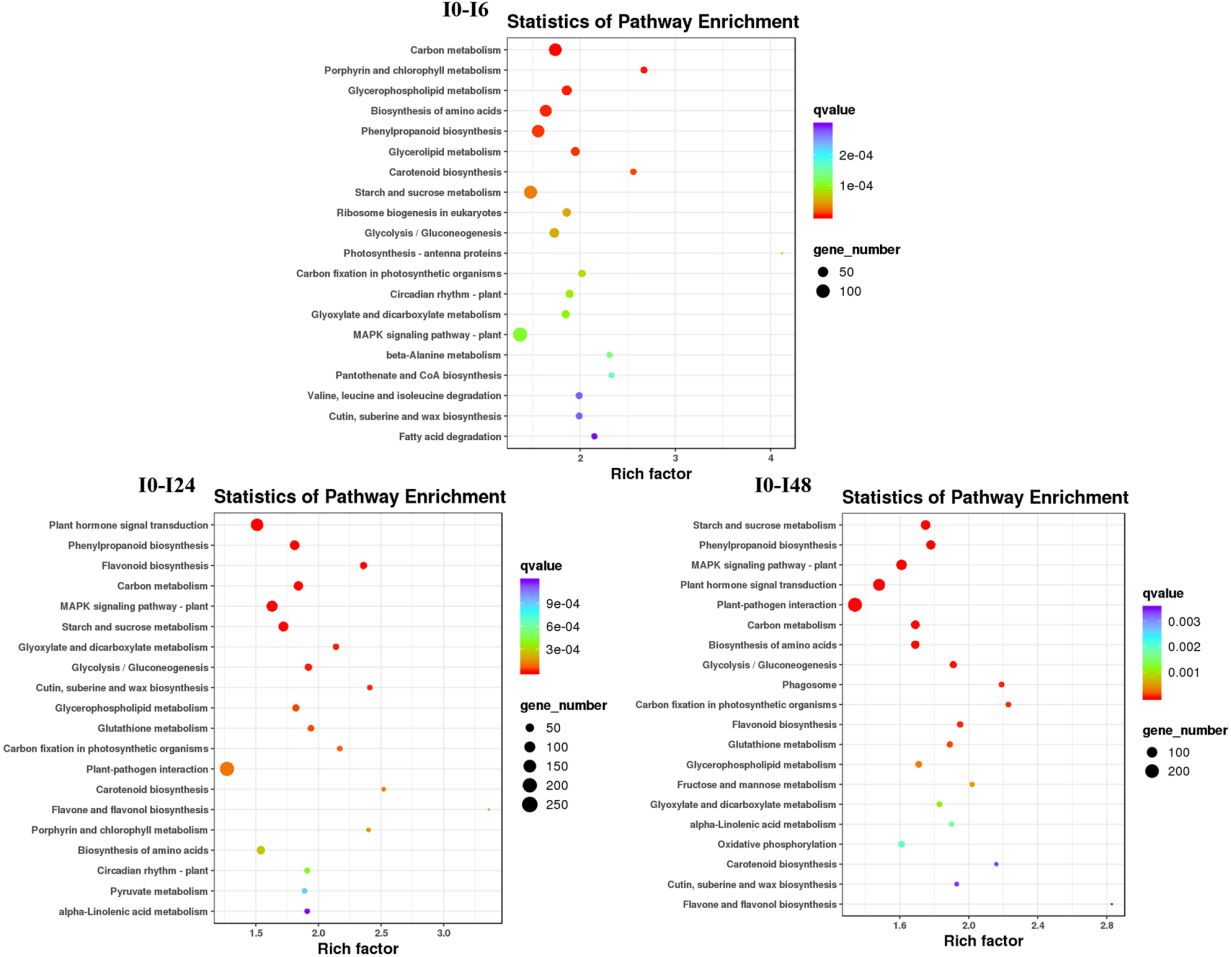
Top-20 KEGG enrichment pathways in IR29 exposed to salt stress for different time periods.

To further understand the metabolic pathways in rice that respond to salt stress, DEGs observed for each treatment of the two rice cultivars were used in a KEGG enrichment analysis. HH11 and IR29 had similar enrichment in KEGG pathways under salt stress (Figs. 7 and 8). Eight metabolic pathways were co-enriched in the six experimental comparisons and included Carotenoid biosynthesis, Glycerophospholipid metabolism, Carbon fixation in photosynthetic organisms, Starch and sucrose metabolism, Biosynthesis of amino acids, Glycolysis/Gluconeogenesis, Carbon metabolism and Phenylpropanoid biosynthesis (Figs. 7 and 8, Tables S6 and S7). Moreover, Starch and sucrose metabolism was the most enriched KEGG pathway for H0-H24, H0-H48 and I0-I48, which enriched 135, 124 and 117 DEGs, respectively, suggesting its role as a key metabolic pathway for response to salt stress by rice plants (Figs. 7 and 8). Flavonoid biosynthesis and Glutathione metabolism were co-enriched KEGG pathways for the H0-H24, H0-H48, I0-I24 and I0-I48 groups (Figs. 7 and 8, Tables S6 and S7). Flavonoid biosynthesis pathway enriched 47, 46, 54 and 47 DEGs for the H0-H24, H0-H48, I0-I24 and I0-I48 groups, respectively (Tables S6 and S7). Glutathione metabolism pathway enriched 53, 46, 45 and 44 DEGs for the H0-H24, H0-H48, I0-I24 and I0-I48 groups, respectively (Tables S6 and S7). Furthermore, the response to oxidative stress GO term were also enriched in the H0-H24, H0-H48, I0-I24 and I0-I48 groups (Figs. 5 and 6). These results indicate that Flavonoid biosynthesis and Glutathione metabolism could be involved in oxidative stress caused by salt stress in both HH11 and IR29.

### Starch and sucrose metabolic pathway response to salt stress

**Figure 9:**
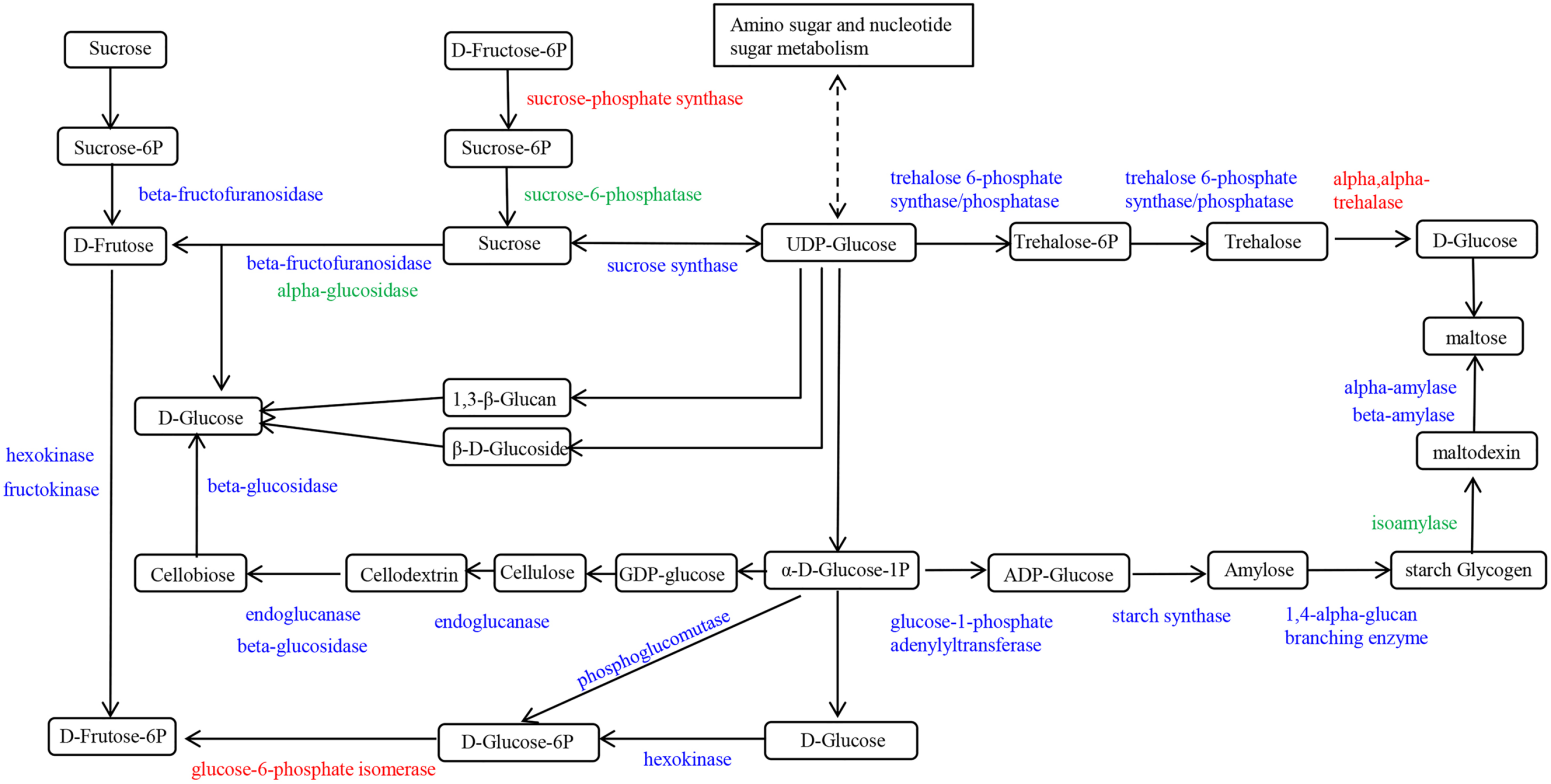
Starch and sucrose metabolism pathway diagram. DEGs that had up- and down-regulated expression in response to exposure of HH11 and IR29 to salt stress are shown in red and green, respectively. DEGs that had both up-regulated and down-regulated expression in the two varieties exposed to salt stress are shown in blue.

**Figure 10:**
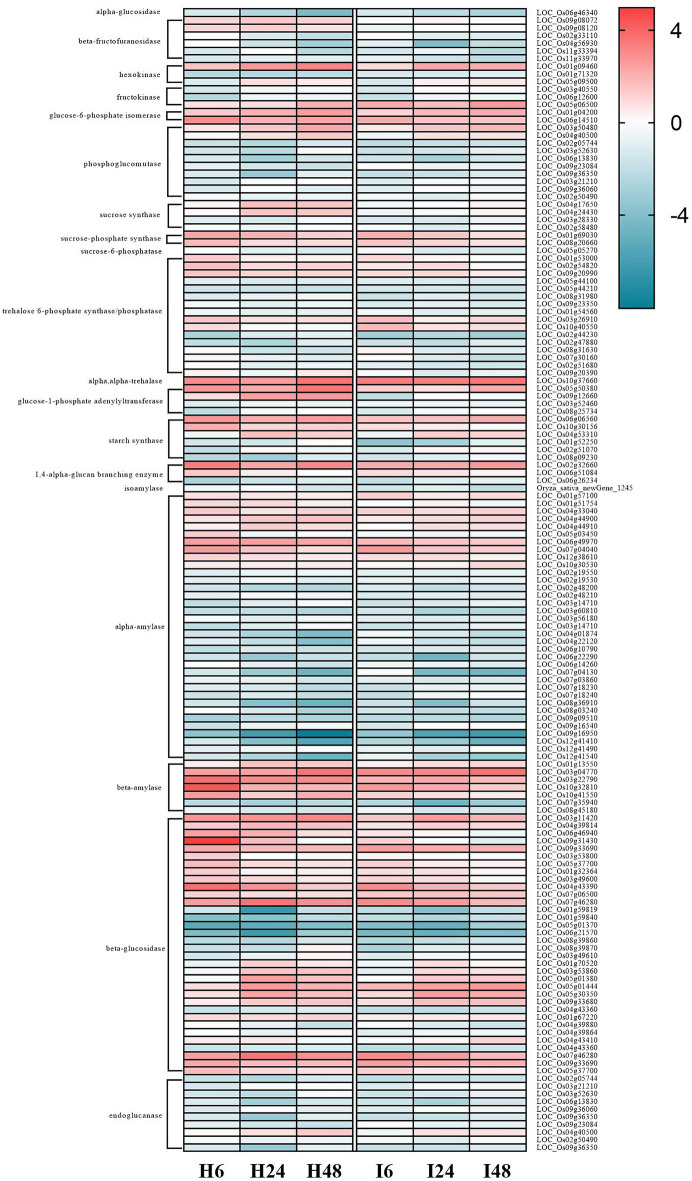
Expression level of DEGs in the Starch and sucrose metabolism pathway. H6, H24 and H48 represent H0 vs. H6, H0 vs. H24 and H0 vs. H48 in the HH11 cultivar, respectively. I6, I24 and I48 represent I0 vs. I6, I0 vs. I24 and I0 vs. I48 in the IR29 cultivar, respectively.

Statistical analysis of the DEGs enriched in the Starch and sucrose metabolic pathway (ko00500) showed that 130, 135, 124, 119, 114 and 117 DEGs were enriched in the H0-H6, H0-H24, H0-H48, I0-I6, I0-I24 and I0-I48 groups, respectively (Fig. 9 and Table S8). A total of 149 DEGs were obtained in the six compared groups after eliminating duplicate DEGs (Fig. 10). The highest number of genes in the pathway were those encoding alpha-amylase, and most of these genes had down-regulated expression (Fig. 10). Meanwhile, seven beta-amylase genes had differential expression with 5 having up-regulated expression and only two having down-regulated expression (Fig. 10). The second-most frequent genes having differential regulation in Starch and sucrose metabolic encoded beta-glucosidase genes, with more having up- than down-regulation (Fig. 10). In addition, 2 glucose-6-phosphate isomerase genes, 2 sucrose-phosphate synthase (SPS) genes and 1 alpha-trehalase genes had up-regulated expression in the H0-H6, H0-H24, H0-H48, I0-6, I0-24 and I0-I48 groups (Fig. 10). Notably, 2 sucrose synthase (SS) genes (*LOC_Os04g17650* and *LOC_Os04g24430*) and a glucose-1-phosphate adenylyltransferase gene (*LOC_Os09g12660*) had up-regulated expression in HH11, but down-regulated expression in IR29, suggesting that they could be candidate genes that regulate salt tolerance (Fig. 10).

### Glutathione metabolic pathway response to salt stress

**Figure 11:**
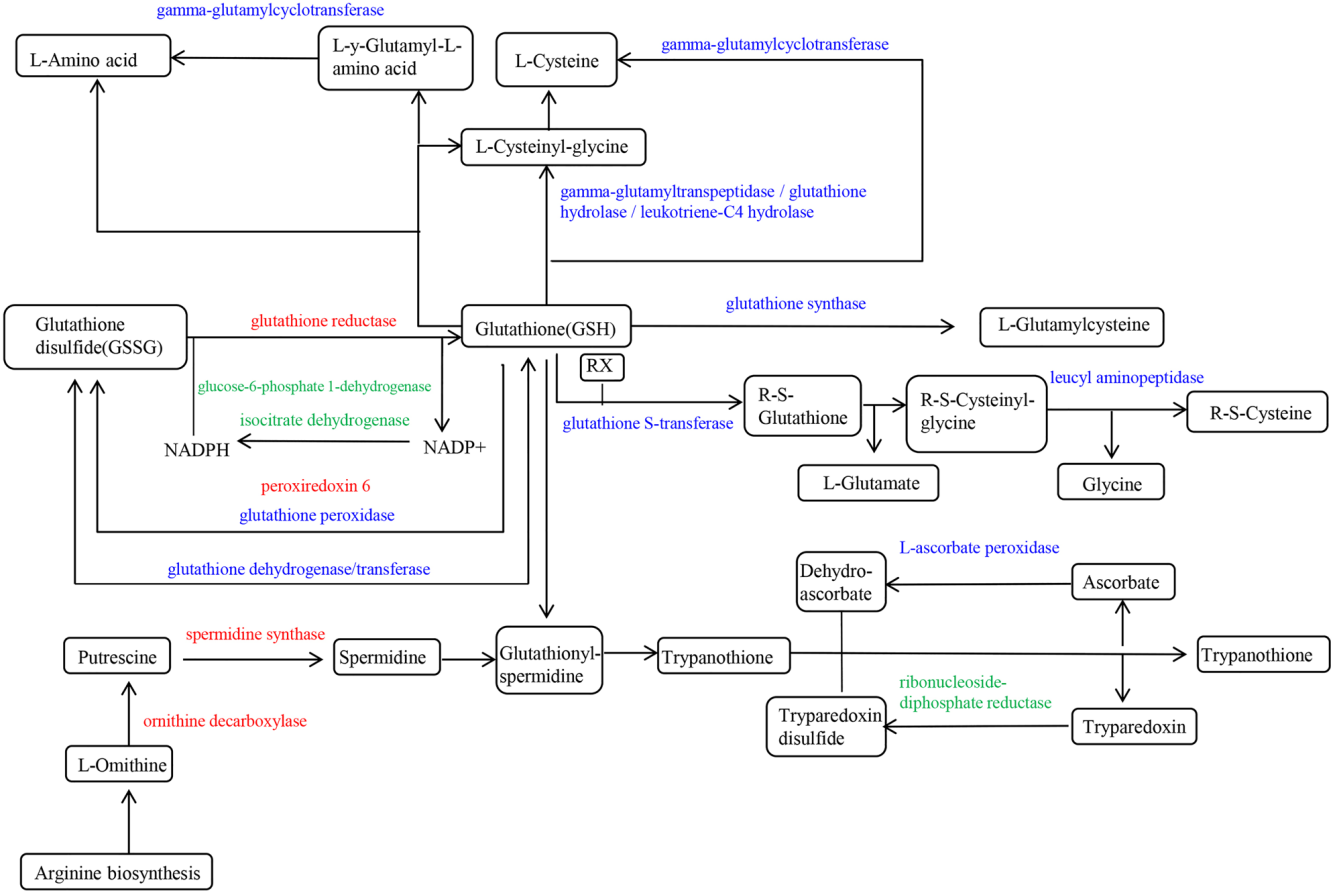
Glutathione metabolic pathway diagram. DEGs that had up-regulated and down-regulated expression in response to exposure of HH11 and IR29 to salt stress are shown in red and green, respectively. DEGs that had both up-regulated and down-regulated expression in the two varieties exposed to salt stress are shown in blue.

**Figure 12:**
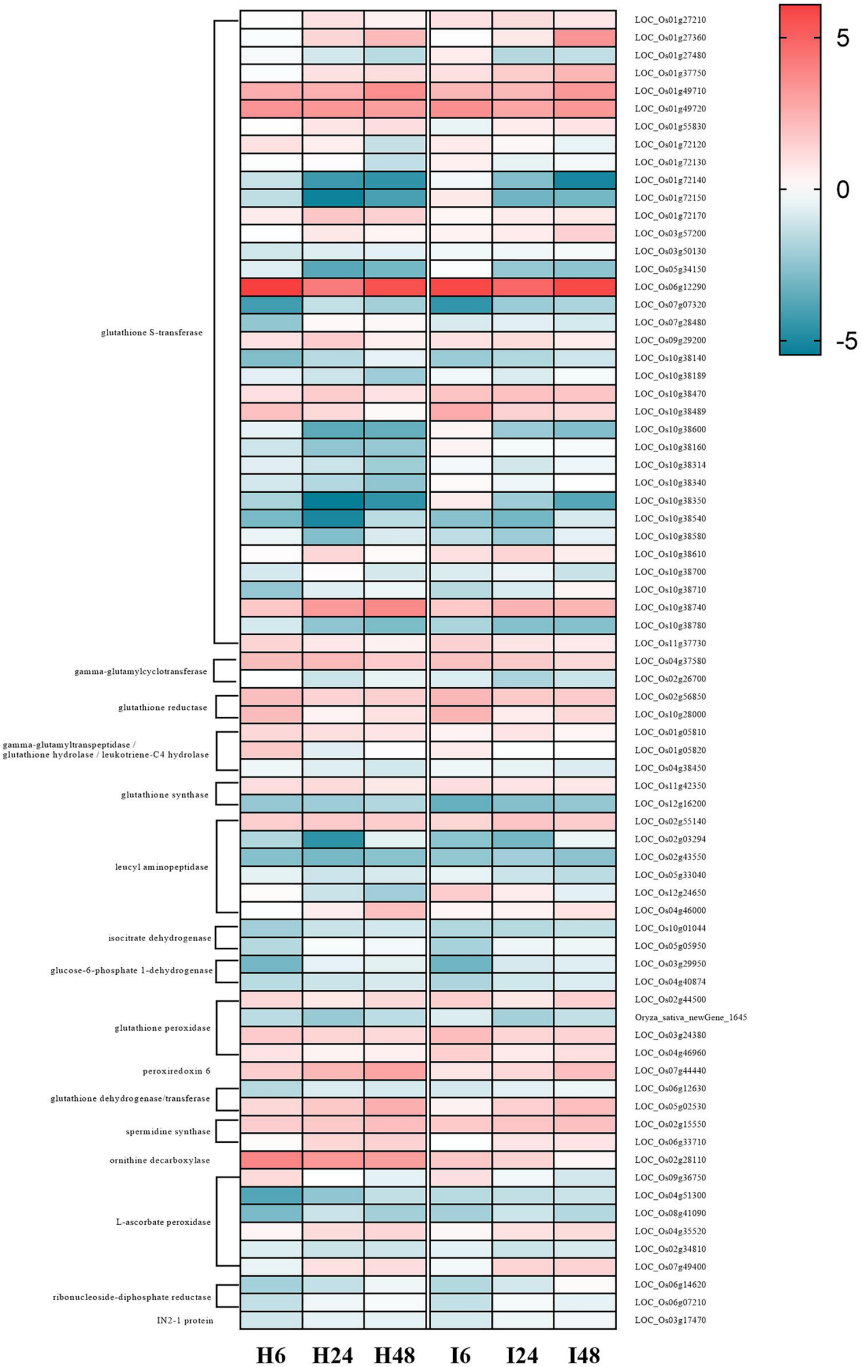
Expression level of DEGs in the glutathione metabolism pathway. H6, H24 and H48 represent H0 vs. H6, H0 vs. H24 and H0 vs. H48 in the HH11 cultivar, respectively. I6, I24 and I48 represent I0 vs. I6, I0 vs. I24 and I0 vs. I48 in the IR29 cultivar, respectively.

Glutathione (GSH) is an important antioxidant and free radical scavenger in plants that can minimize stress-induced damage. KEGG enrichment analysis showed that 47, 53 and 46 DEGs were enriched in the glutathione metabolism pathway (ko00480) for H0-H6, H0-H24 and H0-H48 groups, respectively (Fig. 11 and Table S8). Meanwhile, for IR29, 46, 45 and 44 DEGs were enriched in I0-I6, I0-I24 and I0-I48 groups, respectively (Fig. 11 and Table S8). After summarizing and eliminating redundancy, 74 DEGs were identified for the two cultivars and plotted in a heat map (Fig. 12). Among these 74 DEGs, 36 were glutathione S-transferase (GST) genes (21 downregulated and 15 upregulated), indicating the importance of GST in this pathway (Fig. 12). The most significantly up-regulated GST gene was *LOC_Os06g12290*. The 3 GST genes having the highest degree of down-regulation were *LOC_Os01g72140*, *LOC_Os01g72150* and *LOC_Os10g38350*. Glutathione reductase, spermidine synthase and ornithine decarboxylase genes also had up-regulated expression. Down-regulated genes included leucyl aminopeptidase, L-ascorbate peroxidase, isocitrate dehydrogenase and glucose-6-phosphate-1-dehydrogenase. At a protein level, expression of ribonucleoside-diphosphate reductase and IN2-1 protein were down-regulated under salt stress.

## Discussion

Salt stress has a significant effect on plant growth and development with significant inhibition of seed germination, root length and plant height^35^. In this study, rice plants exposed to increasing time of salt stress showed gradual curling of leaves, yellowing of leaf tips and sagging or collapsing of some stalks. The growth of IR29 (salt-sensitive) was more severely inhibited than HH11 (salt-tolerant). Here we examined the physiological and molecular mechanisms affected by salt stress.

### Physiological response to salt stress by rice seedlings

Salt stress can promote accumulation of reactive oxygen species (ROS) in plant cells. Low levels of ROS can activate signaling pathways, whereas accumulation of excess ROS can compromise cell membrane structures. Lipid oxidation produces multiple secondary products that can aggravate oxidative damage. Malondialdehyde (MDA) is the main product of polyunsaturated fatty acid peroxidation, and is an important index of membrane lipid peroxidation^36^. A previous study showed that MDA content increased in salt-sensitive IR29 seedlings exposed to increasing time of salt stress (6 ds/m ≈ 72 mM and 12 ds/m ≈ 144 mM), but no significant change in MDA levels was observed for salt-tolerant strains^37^. It was different with our results. The reason may be that the NaCl concentration (200 mM) was too high in this study. Moreover, the salt tolerance of different varieties had some differences in the physiological response. The H_2_O_2_ content of IR29 was significantly higher than that of HH11. These results may be associated with the higher ROS scavenging activity or stronger physiological regulation of the salt-tolerant HH11 that allows better adaptation to a salt-stressed environment. The activity of antioxidant enzymes, including SOD, POD, CAT, and APX, is also known to be increased to remove ROS from plant cells^38^. Kordrostami et al.^39^ found that SOD and POD activity of rice varieties with strong salt tolerance was higher or increased more rapidly than that of sensitive varieties. We obtained similar results in this study. The antioxidant enzyme activity of the two rice materials was generally increased by salt stress. The GR and GPX activity of Pokkali (salt-tolerant) were higher than IR64 (salt-sensitive) under 200 mM salt stress^40^. There were similar results in this study. These results indicated that HH11 had stronger antioxidant activity in response to salt stress that resulted in a lower content of MDA and H_2_O_2_ than in salt-sensitive IR29.

Proline accumulation is a common physiological response of plants under various abiotic stresses. The accumulation of proline can reduce proteolysis and also helps stabilize subcellular structures, remove free radicals, and increase redox potential^41^. Higher amounts of proline can also prevent cell dehydration and maintain internal stability of cells to reduce the harmful effects of salt stress. The proline content of rice was previously shown to increase with increasing time of exposure to salt stress, and salt-tolerant varieties accumulated more proline than did salt-sensitive varieties^42^. However, in this study we observed a significantly higher increase in proline content for salt-sensitive IR29 compared to salt-tolerant HH11. This result might be due to a lower osmotic regulation threshold or lower activity of antioxidant enzymes of the salt-sensitive IR29.

### Change in gene transcription of rice seedings under salt stress

Rice plants also exhibit positive physiological responses under salt stress. Here transcriptome analyses were used to explore pivotal salt-responsive metabolic pathways and DEGs. The highest number of DEGs was seen at 6 h of salt stress treatment (7462 DEGs for HH11 and 7566 DEGs for IR29). This number then decreased at 24 h (6363 DEGs for HH11 and 6075 DEGs for IR29) and increased slightly at 48 h (6636 DEGs for HH11 and 6136 DEGs for IR29). These results are consistent with those seen for *Arabidopsis thaliana* under salt stress in which the number of DEGs increased rapidly with 6 h of salt stress, and reached a maximum at 12 h before decreasing at 24 h and increasing again at 48 h^43^. The results here and in *A. thaliana* indicate that gene expression can quickly respond to salt stress (within 6 h) and continues to adapt in the presence of prolonged salt stress. Fewer DEGs had up-regulated expression than down-regulated expression for both HH11 and IR29, which is similar to that seen for DEGs in roots of Nipponbare rice plants at 24 h and 72 h of salt stress treatment^44^. Together these results indicate that salt stress negatively affects rice gene transcription, resulting in down-regulated expression of most DEGs within 24 h of salt stress treatment.

GO enrichment analysis showed that the distribution of enriched DEGs under salt stress was roughly similar between the two rice cultivars. Five main GO enrichment terms for response to salt stress were identified for HH11 and IR29: carbohydrate metabolic process, response to oxidative stress, oxidoreductase activity, metal ion transport and metal ion binding, suggesting that these are important pathways for response of rice plants to salt stress. A previous study that carried out a GO enrichment analysis in rice roots showed that the DEGs were mainly related to protein kinases and calcium-binding, plant hormone signaling and metabolism, transcriptional regulation, metabolic pathways, antioxidant activities and ion transport^44^. Moreover, Khan et al. found that most DEGs identified in tolerant and susceptible rice phenotypes were related to sodium transport, photosynthesis, stress signal, cell redox homeostasis, and heat shock proteins^45^. Together, these findings show regulation of antioxidative activity and maintaining osmotic balance play vital roles in salt stress tolerance in rice.

Enrichment in metabolic pathways of DEGs was further analyzed by KEGG. The KEGG enriched pathways of the two rice cultivars were similar under salt stress, with Starch and sucrose metabolism having significant enrichment in both HH11 and IR29. Starch and sucrose are the main products of photosynthesis, and also provide important carbohydrates to sustain plant growth and development^46^. Meanwhile, Zhang et al. found that expression of several starch and sucrose metabolism-related genes was induced at the seedling stage after salt treatment in all genotypes of rice plants tested^47^. Here we found that the KEGG pathways Flavonoid biosynthesis and Glutathione metabolism, which are closely related to antioxidant activity, were both enriched in the H0-H24, H0-H48, I0-I24 and I0-I48 groups. Jan et al. found that flavonoid accumulation increased the tolerance of rice plants to combined salt and heat stress by regulating physiological, biochemical, and molecular mechanisms^48^. A previous KEGG analysis based on transcriptome and metabolome analysis showed that glutathione metabolism plays a critical role in resistance to salinity in the rice landrace HD961^49^. These results together suggest that flavonoid biosynthesis and glutathione metabolism pathways are important for activity of the salt tolerance regulatory network in rice.

### Regulation of genes involved in sucrose and starch metabolic pathway exposed to salt stress

Starch and sucrose are two of the most common carbohydrates in plants, and they play an important role in energy storage and supply. The mutual conversion between starch and sucrose depends on the growth and development stage of the plant and the influence of external environmental factors. Starch is crucial for mediating plant responses to abiotic stress, including salinity, drought and extreme temperatures^50^. Sucrose often functions as an osmolyte to protect against damage caused by water stress^51^. Sucrose transport and distribution are important processes for maintaining glucose homeostasis under abiotic stress^52^. Here we found that the sucrose and starch metabolic pathway had enrichment in 180 DEGs, and of these, 149 genes were enriched in both cultivars. The enzymes α-amylase and β-amylase catalyze conversion of maltodextrin to maltose, and thus the expression level of these genes affects the amount of maltose (Fig. 10). Here α-amylase genes were the most abundant DEGs, and the only one for which there were more down- than up-regulated genes. Meanwhile, only seven β-amylase genes were enriched, and five were up-regulated and the other two were down-regulated. Zhu et al. isolated the β-amylase gene *IbBAM1.1* from sweet potato and found that drought and salt stress both promoted β-amylase activity and starch degradation^53^. This result is consistent with those of our study, indicating that β-amylase expression is upregulated by salt stress. Here, most of the β-glucosidase genes were up-regulated, and the expression level in HH11 was higher than that for IR29. Hence, rice seedlings affected by salt stress are more likely to synthesize soluble sugar to alleviate osmotic stress and increase energy supply. In Chinese rose exposed to salt stress, increased amounts of glucose, fructose and sucrose and up-regulated expression of *Rc-SS1*, *Rc-SS2*, *Rc-SPS1*, *Rc-SPS2*, *Rc-αA1*, *Rc-αA2*, *Rc-αA3*, *Rc-βA1*, *Rc-βA2* and *Rc-βA3* contributed to a decrease in starch content^54^. In this study, two sucrose-phosphate synthase (SPS) genes had up-regulated expression under salt stress. SPS drives irreversible synthesis of sucrose and is also the rate-limiting enzyme for this process. Here we found differential expression of two *SPS* genes (*LOC_Os01g69030* and *LOC_Os08g20660*) in response to salt stress. Sucrose synthase (SS) is a reversible enzyme that catalyzes both sucrose synthesis and sucrose decomposition. Two *SS* genes (*LOC_Os04g17650* and *LOC_Os04g24430*) had up-regulated differential expression in HH11, but not differential expression in IR29, indicating different responses in salt-tolerant and -sensitive rice varieties. The *glucose-1-phosphate adenylyltransferase* gene (*LOC_Os09g12660*) was also differential expression in HH11. Based on our results, *LOC_Os01g69030*, *LOC_Os08g20660*, *LOC_Os04g17650*, *LOC_Os04g24430* and *LOC_Os09g12660* could be further evaluated for their role in regulating salt tolerance.

### Regulation of genes involved in glutathione metabolic pathway exposed to salt stress

Glutathione (GSH) regulates a variety of metabolic processes in plants, including xenobiotic detoxification, maintaining redox balance, immunity modulation and antioxidant defense^55^. The glutathione metabolic pathway plays an important role during abiotic stress, wherein GSH is often oxidized to GSSG^56^. In this study, 74 DEGs in the glutathione metabolic pathway were significantly enriched, and of these 36 DEGs were glutathione S-transferase (GST) genes. In soybeans, overexpression of *GmGSTU23* was associated with significantly higher GST activity, GR activity and GSH content of transgenic plants relative to wild type plants under salt stress, indicating that enhanced glutathione transferase activity could mediate ROS clearance and glutathione content^57^. Here we found that *LOC_Os06g12290* expression was significantly higher than that of other GST genes, reflecting its importance in responses to salt stress. Meanwhile, expression levels of the GST gene *LOC_Os10g38740* significantly differed between HH11 and IR29. Thus, the role of *LOC_Os06g12290* and *LOC_Os10g38740* in salt tolerance should be further explored since glutathione reductase is an important enzyme that converts oxidized glutathione into reduced glutathione to maintain the content of reduced glutathione in cells. Here, expression of two glutathione reductase genes (*LOC_Os02g56850* and *LOC_Os10g28000*) in HH11 and IR29 had up-regulated expression under salt stress, suggesting that plants need higher amounts of reduced glutathione to scavenge ROS. A previous study showed that rice, sugarcane, corn and chickpea plants had increased glutathione reductase activity in response to salt exposure, indicating that plants up-regulate activity of antioxidant pathways to mitigate oxidative damage caused by salt stress^58–61^. In glutathione biosynthesis, γ-glutamyltranspeptidase plays an important role in the glutamyl cycle by decomposing extracellular glutathione to provide cysteine^62^. The expression of γ-glutamyltranspeptidase decreased with increasing time of salt stress, and may lead to a decrease in the amount of cysteine broken down extracellularly to maintain a certain amount of glutathione. Such activity balances ROS and GSH to minimize stress-induced damage.

## Conclusion

The salt-tolerant rice cultivar HH11 had more favorable adjustment in antioxidant and osmotic activity than the salt-sensitive rice cultivar IR29 upon exposure to salt stress, and in turn HH11 had less growth inhibition than IR29. A large number of DEGs that responded to salt stress were identified through comparative transcriptome analysis in this study. HH11 and IR29 had many DEGs in common, but the gene expression level for salt-tolerant HH11 was higher, likely reflecting enhanced regulatory activity. GO and KEGG analysis showed that HH11 and IR29 had similar enriched pathways in response to salt stress, with enrichment of carbohydrate metabolic process, response to oxidative stress and ion transport. Two *SPS* genes (*LOC_Os01g69030* and *LOC_Os08g20660*), two *SS* genes (*LOC_Os04g17650* and *LOC_Os04g24430*), a *glucose-1-phosphate adenylyltransferase* gene (*LOC_Os09g12660*) and two *GST* genes (*LOC_Os06g12290* and *LOC_Os10g38740*) in the sucrose and starch metabolic pathway and glutathione metabolic pathway were identified in this study as candidate genes that should be further examined to determine their role in responses of rice plants to salt stress.

### Supplementary Information

Supplementary Information.

## Data Availability

The datasets can be found in online database. The raw read data of RNA-seq have been deposited in the GSA (accession: CRA011547), China National Center for Bioinformationer / Beijing Institute of Genomics, Chinese Academy of Sciences (https://ngdc.cncb.ac.cn/gsa/).
