# Supplementary material for: Comparative transcriptome analysis of gene responses of salt-tolerant and salt-sensitive rice cultivars to salt stress
Source: Sci Rep. 2023 Nov 4;13:19065. doi: 10.1038/s41598-023-46389-1 (PMC10625528; doi:10.1038/s41598-023-46389-1)
Supplement: Supplementary file 1 — Supplementary Information. [file 41598_2023_46389_MOESM1_ESM.zip › Supplementary materials-revised/Supplementary table legends.docx]

**Supplementary table legends**

Table S1. Primer sequences for qRT-PCR.

Table S2. RNA-seq data quality.

Table S3. Number of DEGs in comparisons of different groups.

Table S4 GO enrichment analysis of comparisons of different groups for HH11.

Table S5 GO enrichment analysis of comparisons of different groups for IR29.

Table S6 Enriched KEGG pathway analysis of comparisons of different groups for HH11.

Table S7 Enriched KEGG pathway analysis of comparisons of different groups for IR29.

Table S8 Statistical analysis of DEGs in KEGG pathway for comparisons of different groups for HH11 and IR29.
